# Hostage to fortune: an empirical study of the tobacco industry’s business strategies since the advent of e-cigarettes

**DOI:** 10.1080/09581596.2018.1552778

**Published:** 2018-12-18

**Authors:** Marisa de Andrade, Kathryn Angus, Gerard Hastings, Nikolina Angelova

**Affiliations:** a School of Health in Social Science, Counselling, Psychotherapy and Applied Social Sciences, University of Edinburgh, Edinburgh, UK; b UK Centre for Tobacco and Alcohol Studies (UKCTAS), UK; c Group for Research on Inequalities and Tobacco (GRIT), University of Edinburgh, Edinburgh, UK; d Institute for Social Marketing, Faculty of Health Sciences and Sport, University of Stirling, Stirling, UK; e Centre for Tobacco Control Research, Faculty of Health Sciences and Sport, University of Stirling, Stirling, UK; f The Open University Business School, Milton Keynes, UK; g Ecole des hautes études en santé publique (EHESP), Rennes, France

**Keywords:** tobacco industry (TI), tobacco harm reduction (THR), electronic cigarettes (e-cigarettes), electronic nicotine delivery systems (ENDS), business strategies

## Abstract

The tobacco market has been transformed by the arrival of e-cigarettes and array of alternative nicotine delivery systems (ANDS). Public health has struggled to cope with these changes and clear divisions are apparent, but less is known about the tobacco industry (TI) response. This first empirical study to examine TI and independent ANDS companies’ business strategies fills this gap. Primary data were collected through 28 elite interviews with senior/influential TI and independent stakeholders, triangulated with a documentary analysis of company reports, investor analyses, market research, and consultation responses (1022 documents). A deliberately emic analysis shows that tobacco multinationals were initially disconcerted by ANDS, but logic provided by the fiduciary imperative is enabling them to turn a potential threat into profitable opportunities. Interviewees argue market changes played to their strengths: customer links, expertise in nicotine, and enormous financial resources. This enabled portfolio diversification in which combustible and ANDS coexist; providing potential to develop robust scientific and regulatory positions and hope of retrieving corporate reputations. The principal threat for major tobacco players comes from the independent sector, which is prepared and able to satisfy bespoke consumer needs. Multinationals by contrast need to turn ANDS into a genuinely mass-market product appealing to its global customers. They are making progress. Given the continued buoyancy of the combustibles market, they have extensive resources to continue their efforts. Disruptive innovations are not unique to tobacco control. Equivalent technological solutions – with concomitant business opportunities − are emerging in obesity and alcohol fields with implications for public health.

## Introduction

The last five years have seen major innovations in the tobacco and nicotine market (Bauld, Angus, de Andrade, & Ford, 2016). The arrival of the electronic cigarette (e-cigarette) and multiple other alternative nicotine delivery systems (ANDS) means that the traditional cigarette (or ‘combustible’) has been joined by a portfolio of products spanning new categories: first-generation e-cigarettes (closed system cig-a-likes, which replicate the look and feel of combustibles); vapours, tanks, and mods (VTMs) with open refillable systems; tobacco-heated products; and licensed medicinal products (Bauld et al., 2016). While public health has struggled to cope with these sudden changes (Kamat & Van Dyke, 2017; Russell, Wainwright, & Tilson, 2018; Sim & Mackie, 2014), following Milton Friedman’s dictum, ‘The business of business is business’ (Ridgers, 2012), the tobacco industry (TI) has responded with clear and productive focus (Branston & Sweanor, 2015). There has been much speculation about the TI’s business intentions regarding these developments but little empirical evidence.

This study with ANDS industry stakeholders from both tobacco companies and independent producers (with no ties to the TI) fills this gap. Grounded in critical debates concerning the relationship between corporate profit making and public health, it problematises tobacco control’s polarised positions on whether ANDS are ‘good’ or ‘bad’ for public health (de Andrade, Spotswood, Hastings, Angus, & Angelova, 2017). It does this by critiquing an emic analysis of business strategies (from the perspective of interviewees) being deployed in the e-cigarette/ANDS market by both tobacco multinationals and independent companies.

The decision to interview TI representatives was a difficult one for the research team (see below) given the well-documented deceit of these companies and the obvious conflict of interest between the business corporation’s quest for profits and harm to public health caused by smoking. Bakan (2004) argues that this pursuit of self-interest is nothing short of psychopathic, and like their human equivalents, this ruthlessness is hidden beneath a veneer of superficial charm (Hastings, 2012).

Many researchers have warned against the power and influence of the tobacco multinationals and, for example, for the need to focus efforts on controlling them through government action (e.g. see de Beyer & Brigden, 2003) or to guard against them manipulating the ‘definition of the parameters of the problem and the elaboration of solutions’ (Breton, Richard, & Gagnon, 2007, p. 360). Through this lens, we are reminded of the industry’s unhealthy tactics including claims of ‘junk science’ to fulfil political agendas, withholding data adverse to corporate products, using information in misleading ways, corporate funded research, funding researchers and institutions to conduct ‘independent’ studies at arm’s length, threatening to sue (or actually suing), infiltrating scientific groups, creation of doubt to prevent litigation or regulation when it suits, diverting attention from harmful products, dividing and conquering unified groups by feeding controversies, and promoting corporate social responsibility (CSR; Freudenberg, 2014; Grougiou, Dedoulis, & Leventis, 2016; Moodie et al., 2013; Oreskes & Conway, 2010; Wiist, 2010). In this context, seeking the TI’s opinions seems ill-advised at best, and Article 5.3 of the World Health Organization Framework Convention on Tobacco Control (WHO FCTC) – which deliberately isolates public health from industry influence (WHO, 2003) – is a sensible consensus to adopt.

However, the arrival of ANDS has disrupted this unanimity. The dilemmas researchers face in attempting to traverse the boundaries of such a politicised field as tobacco control have become more complicated through a public health community divided over ‘two sides’ of an e-cigarette ‘argument’ that ‘have produced a global divide on policy strategies’ (Green, Fielding, & Brownson, 2018, p. 189). It is increasingly difficult to adopt a neutral stance when conducting and trying to publish e-cigarette research: papers are rejected for not being grounded in a pro- or anti-tobacco harm reduction position (often influenced by reviewers’ and journals’ views on the subject), and there is an a priori assumption, based on their deceitful past, that the TI’s strategies are questionable and that ANDS could be their newest hoodwink (de Andrade et al., 2017; Gornall, 2015).

Other established tobacco control positions have also been disrupted by ANDS. Jacobson, Wasserman, and Raube’s (1993; cited in Breton et al., 2007, p. 360) observation that ‘in the eyes of legislators and individuals, the industry has truly lost the debate on scientific evidence’ seems less convincing in an era of harm reduction and disruptive new product development (Hasselbalch, 2016). The monolithic conception of ‘Big Tobacco’ as a homogeneous entity has been undermined by the arrival of independent companies with no TI connections promoting ANDS, and there has continued to be ‘an ironic convergence in the tobacco industry and [parts of the] tobacco control community positions’ (Mair & Kierans, 2007, p. 104).

At the same time, public health cannot ignore the ‘commercial determinants of ill health’ (Hastings, 2012) and that tobacco-use continues to be the most hazardous of these. Studying the disease vector is, therefore, vitally important, and interviewing industry representatives is, arguably, a crucial part of this. Furthermore, if public health does not do this direct research, others will, and an important opportunity will be lost. As Hastings (2012, p. 3) argues: ‘Public health has to demand a place at the macroeconomic table; it has to contribute to the debate about where corporate capitalism is going and ensure that the public health implications of business decision making are fully appreciated’.

## Materials and methods

The original funding proposal did not include primary data collection with representatives from the TI, with applicants citing Article 5.3 of the WHO FCTC. A conditional offer response from Cancer Research UK’s Tobacco Advisory Group Committee noted ‘that the proposal would benefit from speaking to people responsible for marketing the products themselves, or creating their marketing strategies’ and ‘felt the applicants could use intermediaries to achieve this if they felt this was necessary’. After careful consideration, the research team decided to conduct the interviews themselves.

An emic analysis was conducted using both primary data from interviews and a documentary analysis of the business strategies being deployed in the ANDS market by tobacco and independent companies. This qualitative approach was an attempt to understand and explore companies’ motivations, intentions, and strategies from the subject’s perspective – using insider accounts (Headland, Pike, & Harris, 1990). Thoughts and actions were collected and critiqued in terms of the actors’ (both independent stakeholders’ and the TI’s) culturally and historically bound self-understanding (Morris, Leung, Ames, & Lickel, 1999).

Primary data were collected through a unique data-set of elite interviews (Berry, 2002; Welch, Marschan-Piekkari, Penttinen, & Tahvanainen, 2002). The elite interviewee is

an informant … who occupies a senior or middle management position; has functional responsibility in an area which enjoys high status in accordance with corporate values; has considerable industry experience and frequently also long tenure with the company; possesses a broad network of personal relationships; and has considerable international exposure. (Welch et al., 2002, p. 613)

Interview data were triangulated against a documentary analysis of more than 1000 documents to understand what views senior stakeholders were expressing, to whom (e.g. investors, media, or retailers) and why. Hasselbach (2016) investigated stakeholders’ framing of the European Union (EU) policy debate on e-cigarettes regulation; however, the authors could not locate any empirical studies adopting a business perspective.

### Primary interview data collection

The lead researcher collecting, and principally analysing, the primary data was a female academic researcher and qualified investigative journalist with extensive interviewing experience. She is part of a group that has critically analysed e-cigarette marketing and industry involvement in public health policy (de Andrade, Hastings, & Angus, 2013; Hastings, de Andrade, & Moodie, 2012). This remains a highly contested area for public health research, and the study purpose was not to pitch a pro- or anti-harm reduction perspective but to explore business strategies from the viewpoints of business stakeholders. As far as possible, the interviewees’ own words are used.

A purposive sampling strategy was used to identify appropriate individuals and gatekeepers among TI stakeholders and independents. To facilitate access, the researcher attended two conference(s)/summit(s) where TI and non-TI stakeholders were present. The purpose of attendance was not to gather data but to meet gatekeepers and potential interviewees and gain further understanding of business strategies. Snowball sampling was used to access additional senior and influential interviewees, through introductions or referrals, until sufficient people had been interviewed. Interviewing continued until saturation was reached.

Despite a predictable reluctance to talk about such a controversial topic, it was possible to achieve this because the TI is so monolithic: just five transnational tobacco companies control 84% of the global cigarette market (Campaign for Tobacco-Free Kids, 2016). Twenty-eight individuals were interviewed, split into TI (interviewee has or is working for/with TI as employee/consultant/analyst, *n* = 13) and non-TI interviewees (stakeholders in the e-cigarette sector who are independent from the TI including manufacturers/distributors/retailers/advocates, *n* = 15; see de Andrade et al., 2017, for interview settings and duration). TI interviewees were or had been associated with three of the big five tobacco multinationals and/or other major tobacco companies as employees, consultants, or analysts. This partial coverage may be a source of bias. Independent respondents stipulated that they had no ties with the TI (financial or otherwise) and as such might have had different interests and motivations. The researchers have no personal or professional relationshipto any interviewees, but some had previously attended the same tobacco control meetings/conferences and/or were aware of their research.

An open-ended interview style drew on the aims of the study, which were to examine tobacco companies’ business approaches, targeting strategies, positions on harm reduction, and marketing plans, and was shaped by interviewees’ responses. The topic is extremely contentious and subject to a great diversity of opinion, so the interviewer sought to maintain a neutral position. Interviews were conducted between April 2015 and November 2016 either face to face or by telephone. Participants received a project information sheet and informed consent form and had the opportunity to ask questions about the study. Interviews were recorded on digital audio files, given an anonymous filename and professionally transcribed. These transcriptions were subsequently checked by the research team. Transcript storage was on secure, password-protected servers and encrypted.

Ethical approval was granted by the School of Health in Social Science Research Ethics Committee at the University of Edinburgh.

### Documentary analysis

It can be difficult to access data in most corporate sectors that are likely to give an insight into a company’s defined business objectives, scrutiny of its operating environments, and coordination of its long-term bearing. ANDS stakeholders operate in a competitive environment and are likely to withhold long-term plans for commercial sensitivity reasons in order to seek advantage over rival companies. Documentation likely to contain business strategies are company reports, analyses prepared for investors, press releases, newspaper articles, and third-party market research reports. Databases of internal TI documents have been researched previously (e.g. see Peeters & Gilmore, 2013, 2015) and were excluded from this study. A comprehensive literature search captured publicly available English-language data from multiple sources: databases, websites, and print sources (see Appendix 1 in Supplement for details). Search terms for the databases included the following: ‘electronic cigarette’, ‘e-cigarette’, ‘vaping’, ‘vapo(u)r device’, ‘business’, ‘strategy’, ‘forecast’, ‘analysis’, and brand names. We note that TI companies are generally publicly listed so there are many reports available, but many of the companies producing ANDS are smaller, and not all are publicly listed. As annual reports were only a small part of our documentary search, this is not a significant source of bias. An initial search was conducted between November 2015 and February 2016 resulting in 1022 uploaded to NVivo for coding. Additional ad hoc searches were conducted for triangulation with primary data until February 2017.

A further data source for triangulation was data extraction of TI and non-TI responses to the Scottish Government’s 2014–2015 consultation on electronic cigarettes and tobacco control (Scottish Government, 2015). From 170 responses, 14 were identified as relevant to this study (linked to ANDS companies; not members of the public/public health; see Appendix 2 in Supplement). Government consultation responses can also provide insights into how companies operate (Ulucanlar, Fooks, Hatchard, & Gilmore, 2014).

### Data analysis and synthesis

The constant comparison method of qualitative analysis was employed to compare new data with previously collected data across three data-sets using a team of researchers to ensure reliability, validity, and minimise bias (see Appendix 3 in Supplement for the coding strategy). Informed by inductive techniques of grounded theory, open coding was used by two researchers for each data-set to identify first-level concepts followed by thematic coding with a clear focus on business strategies. Coding was checked by a third researcher. Analytic meetings were held throughout data collection and analysis to discuss emergent themes, definitions, resolve discrepancies, and reflect on data interpretations as they arose. These informed ongoing interviews conducted by the lead researcher so that interviewees could respond to emergent findings in an iterative way (see Appendix 4 in Supplement for the codes used in the data analysis).

Upon completion of primary interview data collection, a thematic network was created by the lead researcher ‘to explore the understanding of an issue or the signification of an idea [TI and non-TI business strategies], rather than to reconcile conflicting definitions of a problem’ (Attride-Stirling, 2001, p. 387; see Appendix 5 in Supplement for the thematic network). This guided triangulation with documentary sources, substantiated by further ad hoc searches in stage two databases conducted from March 2016 until February 2017 and by market reports/documents sent to the lead researcher during data collection. As this is a rapidly evolving field, this approach ensured findings reflected latest developments.

Given the study’s commercial focus, and its emic approach, final synthesis of the TI’s response to ANDS employed the classic business framework of SWOT analysis, widely recognised in the strategic planning literature to map internal strengths (S) and weaknesses (W) against external opportunities (O) and threats (T); (Helms & Nixon, 2010).

Methodological limitations of this study include the research team’s expertise in researching the impact of marketing and business on society. The first three authors are known in the field of tobacco control and public health for their critical views on industry tactics. Their backgrounds may have influenced the study even though the intention was to maintain a neutral position. This potential bias was minimised by the ability to recruit TI stakeholders for primary interview data collection despite previous public criticisms and including a fourth researcher in the team from an unrelated discipline to assist with data analysis. The unstructured nature of interviews could also be viewed as a limitation, though it is also a strength as it allows for flexibility of responses in a constantly evolving competitive market with stakeholders expressing divergent views. The project was not resourced to do further documentary data searches (e.g. other UK consultations) for longer time periods.

## Results

**Figure 1.**
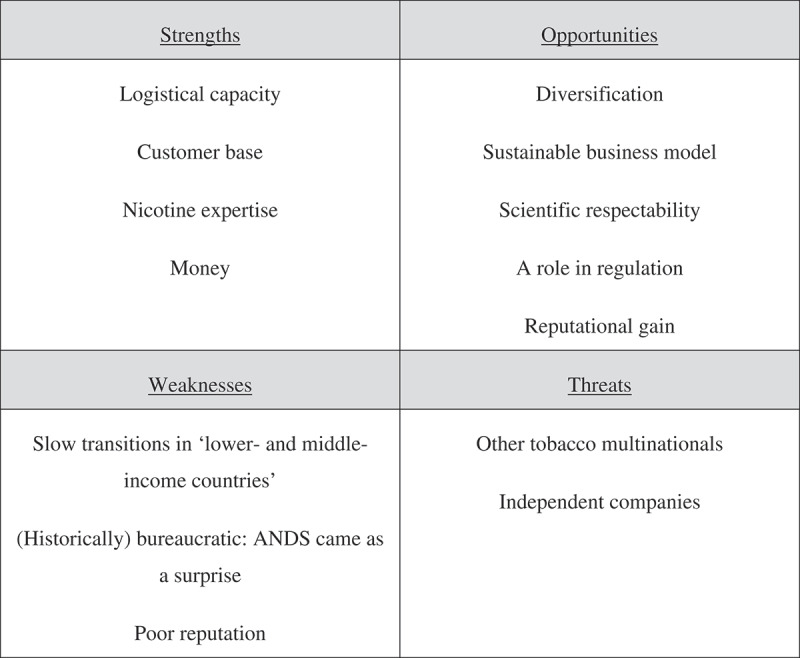
SWOT analysis of the tobacco industry’s response to alternative nicotine delivery systems.

Several TI respondents noted that the tobacco business is driven by the fiduciary imperative: the need to prioritise shareholder returns above all other concerns (Bakan, 2004). This means that the massive changes in the market brought about by the arrival of ANDS are ‘not complicated’, as a TI representative noted, but actually ‘quite easy’ to address because ‘every activity going on is there to please the shareholder … as long as shareholder value is threatened, [the TI’s] gonna continue to protect it’ (Int6). A further TI spokesperson added that the challenge of change is, therefore, reduced to questions about how ‘to grow our market share’ and ‘margins per thousand’, and so turn a possible threat into ‘quite the business opportunity’ (Int15). See our analysis in Figure 1.

### Strengths

According to industry, the TI’s long-established position in the market brings four important strengths. First, it has enormous logistical capacity in production, distribution, and marketing. One TI representative commented on how the ‘sheer size’ of their distribution channels can facilitate growth in this new market as ‘major companies have great distribution networks’ (Int9). This, according to a TI competitor, gives tobacco companies a ‘big edge’ (Int15). British American Tobacco (BAT)-owned Nicoventures echoed this in the documentary analysis by saying that ‘suppliers remain keen to harness the traditional grocery channels to move their products’ (Hegarty, 2015, p. 52).

Independent stakeholders reflected on how this provides an opportunity for e-cigarettes to ‘be connected directly into their [TI] network of distribution, their network of sales’ (Int13). One explained that the TI

followed into the vapour space with their various cig-a-like products that are sold through the same marketing channels of distribution that they already control, which is the convenience stores and the supermarkets and the gas stations. Wherever you find tobacco products, that’s where you find Big Tobacco’s vapour products. (Int19)

This, according to a different independent advocate, means they have the capacity to scale up and distribute these new products – to ‘mass manufacture them’ and then ‘eventually get to the point where you can be profitable’ (Int1).

The TI’s second strength is its customer base, which an industry insider described as a vital asset when ‘business strategy is driven by consumer needs’ (Int11). Int4, a TI executive, said that ‘the people that are using e-cigarettes are the people that used to use cigarettes. So the consumers are the same, at least at the moment’. The interviewee went on to explain that it is, therefore, ‘an absolute natural notion that you would go and start producing e-cigarettes when your consumers have left your product category and gone to another product category’ – ‘that’s just basic common sense’ (int4). Given that the global pool of smokers is about a billion strong, this was framed as a major advantage for industry.

The TI’s third strength, again from the perspective of tobacco representatives, is its extensive technical knowledge about all things nicotine related. One TI executive said that industry is in a ‘better positioned to deliver on the science, to deliver on a product that meets the needs of consumers’ as it has ‘got folks that have done nothing but study tobacco their entire careers’ (Int8).

Finally, given the continuing profitability of combustibles, the industry has financial resources needed to innovate and adapt. Protecting the cigarette market is, therefore, ‘a near term necessity’, according to one TI interviewee, as ‘the stock price and investor interest’ comes from maintaining ‘traditional products … combustible cigarette products, for as long as possible’ (Int9). Another TI interviewee added, ‘all that investment and that innovation costs a lot of money that is funded today by profits from the combustible brand’ (Int8). Commenting on ‘the realities of the current combustible market’ and the fact that ‘cigarettes are obviously a profitable product’, the same interviewee said that ‘simply stopping making those products doesn’t put us in a position to deliver innovation and to get through these costly [regulatory] pathways that are now in front of all of us’ (Int8). A different TI spokesperson further explained that tobacco companies may see traditional tobacco as a ‘sunset business’, but they ‘can’t just stop producing cigarettes, because cigarettes – the income from cigarettes – is the boiler-plate for investment’ (Int6).

Thus, combustibles will ‘remain the mainstay of BAT’s commercial delivery for a long time’, because they provide ‘the funding required to develop less-harmful next-generation products’ (Tuinstra, 2015, p. 24) a sector in which it aims ‘to achieve global leadership … by 2020’ (Rossel, 2016, p. 18). Similarly, Philip Morris International (PMI) notes that ‘while combustible cigarettes are likely to remain at the core of PMI’s business for years, the company has been investing heavily in next-generation products’ with ‘tobacco harm-reduction objectives and commercial objectives starting to align’ (Tuinstra, 2015, p. 16). The company aims ‘to lead the combustible product category, leveraging the great strengths of [its] existing business’ and, like BAT, ‘become the undisputed leader of the Reduced-Risk Product category’ (PMI, 2016a, p. 1). It feels ‘very well positioned to deliver on both growth engines’ (PMI, 2016b, p. 2)^1^.

### Weaknesses

Analysts note, however, that transitions will be slow in ‘lower and middle-income countries’, which ‘are home to 80% of the global smoking population’ (TechNavio, 2016). One TI interviewee explained that ‘emerging markets will be a solid combustible cigarette consuming base for many, many years to come’ (Int9). They went on to clarify that ‘cigarettes are extremely cheap’, so ‘these are much more price driven decisions’ for consumers: ‘it’s really hard for me to imagine a bunch of very low-income individuals … wanting to switch, or even caring, if they can afford cigarettes’ (Int9). The ‘economics of demand’ (Int9) present ‘a dilemma’, which a TI competitor explained as follows: ‘the price point to get into [ANDS] … is still high cos you’ve got [to buy] a device, so how d’you make that economical for the [developing] world?’ (Int4).

The TI’s established position brings two significant further weaknesses, according to independents and TI representatives. First, like many large businesses, tobacco companies had become bureaucratic and set in their ways. ANDS took them by surprise, as this independent interviewee explains:

[ANDS] got on the market without asking permission … so quickly that they weren’t able to be killed off … [The TI] did not start this business. This business was foisted on them by entrepreneurs out of China, picked up by entrepreneurs elsewhere – they reacted, they didn’t create it. (Int1)

A TI spokesperson also spoke about how the TI ‘sat back and watched’ the ‘completely consumer driven revolution’ and only belatedly ‘jumped in the game’ (Int8) when, according to one independent, they became ‘aware that this nascent interloper could cause a threat’ (Int13). A TI executive added that ‘it took some time’ for industry to recognise ‘a space that might become quite big, going forward’ even though ‘vape shops [were] popping up’ and e-cigarettes were becoming a ‘cult thing’ (Int10).

An independent interviewee said the TI had a realisation that ‘every time somebody switches from smoking to vaping [tobacco companies] lose a customer’, so this meant that they needed ‘to be in the game’, to ‘take a place in this market’, and ‘make money from that’ (Int25). TI competitors added that this was a ‘big signal to companies’ that ‘adult smokers are looking for alternatives’ (Int15) as ‘consumers don’t wanna be dying from the products that they’re consuming’ (Int9). As Int8 put it, the TI’s ‘licence to operate is threatened if people view … nothing but malicious intent’.

This points to the TI’s second great weakness: a reputation for ruthless business practice. Several independents noted that ‘nobody trusts the tobacco industry’ (Int19). The lethal characteristics of cigarettes combined with past duplicity have made it a pariah (Christofides, Chapman, & Dominello, 1999; Hastings, 2012).

### Opportunities

Turning to the external environment, ANDS present the TI with opportunities in five areas. First, they enable diversification. For 150 years, the industry has been dependent on one increasingly embattled offering: combustible tobacco. TI executives said that ANDS offer the chance to move to a much stronger ‘portfolio’ business model (Int4). One TI interviewee said that this approach is ‘really important’ as smokers are not a ‘homogenous group’, so ‘there is never gonna be a silver bullet which just works all across all of the different markets in terms of what every single adult smoker is looking for’ (Int15). They highlighted the need for ‘a range of products to satisfy consumer needs’ (Int15), echoing views in tobacco trade press about ‘catering to all consumer needs’ (Rossel, 2016, p. 18).

Thus, for BAT, ‘differentiated products’ with ‘brand strength’ are part of the ‘commercial attractiveness’ of next-generation productions (Wheaton, 2015). It has ‘developed a portfolio of products spanning three categories: tobacco heated products (THP), e-cigarettes and licensed medicinal products’ (Rossel, 2016, p. 18). Similarly, JTI has ‘expanded’ its ‘product portfolio by entering in[to] new categories’ through, for example, the acquisition of the independent company E-Lites to ‘complement’ the company’s ‘heat-not-burn offering’ (McCoy, 2015). Meanwhile, PMI notes that ‘a comprehensive portfolio approach to the category and [its] platforms are designed to address a range of different audiences and usage occasions’ (PMI, 2016a, p. 11).

ANDS also offer long-term strategic opportunities, presenting a ‘sustainable business model’ according to a TI executive (Int9). They added that at present, ‘companies aren’t particularly rewarded or acknowledged for these alternative products that they’re investing in, either through R&D or acquisition’, but these are viewed ‘as necessary for longer-term sustainability’ (Int9). The TI is investing in these products because ‘that’s where [it] see[s] demand is headed, even if it’s taking a little bit longer to catch up, potentially, than originally thought’ (Int9). A TI competitor agreed that ‘it’s all about investment. This is not about putting the products on the shelf, intending to start to make millions or billions from it overnight’ (Int4). A different TI spokesperson added that what ‘we’ll see over the next five years is a flip, where the margins are much much greater than even our most profitable cigarette brand’ (Int8).

This future profitability will be strongly influenced by taxation policy, with heavy tax on combustibles making ANDS more commercially attractive. According to an independent interviewee, the TI has worked out that ‘you’re looking at a much higher level of profit from e-cigs’ as ‘there is no punitive taxation’ on them (Int12). They added,

for once … the retailers, the distributors and the tobacco companies are the biggest stakeholders, as is the case in most other businesses … Now, it doesn’t take a great intellect to see that if you retain more of the selling price … you’re going to be more profitable [and] if they’re gonna be more profitable flogging e-cigs and the environment is made easier for them to do that, then why wouldn’t they? (Int12)

ANDS also offer the TI an opportunity to own safety and science. The ANDS market has had quality control problems, which the TI says it can resolve. It can impose ‘quality standards’ and ‘tighten up’ design to eliminate problems such as ‘exploding batteries’ (Int15). More profoundly, it sees itself as ‘leading the field of the science’ (Int18), citing ‘the large body of [TI-funded] studies that has been published already and that are in the publishing process’ as ‘fundamental’ for business transformation (Int18). According to TI executives, the science is ‘extremely high quality’ ‘because it faces very, very, robust scrutiny and it’s done with very high quality labs, very high quality methodology and it’s published in good quality journals that are not specifically on tobacco’ (Int25). BAT has ‘a new scientific framework to assess the reduced-risk potential of nicotine and new tobacco products’ being developed using a ‘four-stage process’ (Hedley, 2015). Similarly, ‘PMI Science’ has been established to ‘conduct rigorous and scientific assessment to demonstrate that [ANDS] reduce risk and comply with emerging standards’ (https://www.pmiscience.com). This was described by TI representatives as ‘a paradigm shift for our industry’ as ‘no one’s had these types of products before and certainly not with the level of scientific substantiation and evidence which [the TI is] compiling’ (Int18). As a TI spokesperson explained, ‘the one thing that we have, and it’s a business strategy, is that we steward these products substantially’ (Int4).

This stewardship provides a fourth opportunity: a route into regulation. TI respondents said that it is now possible to work with ‘the regulators … and enlighten them of the risks’ (Int11) with ‘millions of pages of high quality scientific research’ (Int18). One TI executive further explained this business strategy:

This is where we would like to … work with … public health, with regulators who have an interest in reduced risk … to find out what is the appropriate regulatory path … So we are investing a huge amount of time, resource, expertise, in the science to enable those conversations and to objectively look at the best route forward. (Int18)

They added that studies are being conducted ‘to demonstrate that they [ANDS] reduce risk’, and these findings will ‘become a corner-stone’ of regulatory applications (Int18). While costly, ‘shaping future regulation’ in this way, as BAT expressed it, is vital (Wheaton, 2015). As a TI spokesperson explained, regulation plays ‘a very outsized role in … the market’ as it ‘governs the rules’ of how the TI ‘can market their products and what types of products and what goes into products’ (Int9).

The focus, according to independent and TI respondents, is still on profitability. One TI interviewee reflected on how tobacco ‘companies are amoral. They’re not immoral. They’re not moral. And if they’re behaving in a moral way, it’s because they believe that’s gonna advance their business interests’, so ‘the health benefits are a pleasant but a, kind of… accident, if you will’ (Int2). A different TI spokesperson added that while there is an ‘element of “we have to do what’s the right thing to do” … you can’t do that at the expense of shareholders’ as you’re ‘running a commercial business’ (Int7). The same proviso applies when BAT’s MD of next-generation products speaks of a ‘win-win-win situation – a win for society as public health aims are advanced, a win for consumers as exciting new products become available and a win for share-holders as sustainable value is generated’ (Tuinstra, 2015, p. 24). So, contributing to public health, or appearing to do so, becomes part of the business strategy. TI industry responses to the Scottish Government’s Consultation on e-cigarettes in 2014/2015 exemplify this strategic approach (see Appendix 2 in Supplement).

Finally, ANDS offer the opportunity to rebuild corporate reputations and guard against litigation. Openness, particularly about risks and addiction, with both stakeholders and the public, is now a key strategy. TI respondents spoke about how the industry has ‘opened up’ ‘the information flow into their R&D process’ (Int9) and are now ‘openly talk[ing] about the amount of money that we [they] invest every year in R&D into the products’ (Int4). Public health was encouraged to look at R&D expenditures to see the increases. Similarly, according to TI executives, companies want consumers to ‘be aware of the risks’ (Int11) and ‘are encouraging retailers to educate staff to be able to deal with the increasingly complex shopper requirements’ (Hegarty, 2015, p. 52). This frankness, according to a TI representative, will bring reputational benefits: ‘if you’re allowed to actually start communicating about risk in a proper way, I think that will lead to a shift [in company standing]’ (Int6).

BAT, for example, wants ‘to lead the segment’ by offering consumers ‘a choice of products across the risk continuum, including vapor, tobacco-heating and licensed medicinal products’ (Tuinstra, 2015, p. 24). In this way, the decisions about risk are delegated to the consumer, making litigation less likely.

### Threats

Now, the TI is established in the ANDS market, the principle threats come from two sets of competitors: other tobacco companies and independent operators. The former is nothing new as the tobacco multinationals were fierce rivals long before ANDS emerged.

The threat from the independents, however, is novel and relates to product characteristics. As one independent explained, for the tobacco companies, the cig-a-like ‘in many respects is the perfect product’ because it is easily mass produced, has ‘low inventory [and] next to no shelving cost’, and is ‘very, very high on margin’ (Int3). It also enables them to exploit their formidable distribution networks. The trouble is ‘it’s just not perfect for the consumer’ (Int3). A TI executive agreed that cig-a-likes are ‘pretty ineffective in terms of giving smokers what they want’ (Int4).

Independents explained that users prefer the more flexible option of buying one ‘bit of hardware’ or tank which they can refill with ‘somebody else’s liquid that you fancy – and there are thousands’ (Int5). While some companies are happy to operate on this small-scale bespoke basis, for a big player, ‘there’s no long-term big commercial market in e-liquids. It’s a commodity product’ (Int5). Furthermore, ‘there’s no barrier to entry into that market’ (Int5), threatening the current market dominance of the big five tobacco companies. The ‘Apple model’, according to independents, may provide a way out here: the TI is working on tank systems which tie customers into using only their refills and components, as happens with the iPhone ‘because that’s a viable business model’ (Int5). There is now an ‘emerging sub-category of closed capsule tank products which tobacco companies in particular are making central to their e-cigarette product pipelines’ (MacGuill, 2015).

## Discussion

The results are presented from the perspective of the respondents, all of whom are closely involved with the tobacco and ANDS markets, and so need to be interpreted with caution. It is also important to note that the TI is not a monolith. It comprises a number of multinationals who are in fierce competition with one another and the recently arrived independents, and this makes generalisation hazardous. Nonetheless, it does seem clear that after the initial shock created by the unexpected arrival of e-cigarettes and ANDS, which at one point might have represented an existential threat to their existence, the tobacco multinationals are doing all they can to turn this potential menace into a range of business opportunities. Interviewees argued that the new arrivals play to their strengths, especially their customer links; expertise in nicotine; and, thanks to combustibles, massive financial resources. This, it was claimed, has enabled them to create multiple opportunities. Specifically, they have been able to diversify into broader product portfolios in which combustible and alternative products can comfortably coexist, set new long-term strategic goals, develop scientific and even regulatory capacity, and to possibly retrieve their corporate reputations by seeming to align with public health objectives. By the same token, ANDS, the interviews suggested, have the potential to reduce the TI’s existing weaknesses, especially its poor standing among both consumers and stakeholders. The principal threat for the major tobacco players comes from the independent sector, which is prepared and able to satisfy a bespoke set of consumer needs. The multinationals by contrast need to turn ANDS into a genuinely mass-market product, which appeals to its global customer base. They are making progress on this and, given the continued buoyancy of the combustibles market, have extensive resources at their disposal to continue the effort.

The study has limitations. Qualitative methods make generalisation difficult and resources inevitably constrained data collection. Furthermore, it measures reported not actual behaviour, and so, as with public surveys, there may be a tendency on the part of interviewees to over-rationalise, on one hand, and understate contradictions, on the other hand. However, as the first empirical investigation with business stakeholders since the advent of ANDS, it gives unique insights into how a market which has such immense implications for public health may develop.

## Conclusion

The public health literature is replete with warnings about the duplicity and malevolence of ‘Big Tobacco’. That is why Article 5.3 of the WHO FCTC explicitly protects tobacco control policies from the ‘commercial and other vested interests of the tobacco industry’. Over 180 countries have now signed up to the treaty, covering 90% of humanity (WHO, 2018).

As Bell (2013, p. 38) notes, however, this can constrain critical thinking:

over the past two decades tobacco studies scholars have had to exercise increasing care in how they frame their research and those whose tone is critical of mainstream tobacco control open themselves up to accusations of alignment with ‘pro-tobacco interests’.

These tensions have been exacerbated by the arrival of ANDS, the pros and cons of which can lead to peer review pressures and are difficult to discuss without being accused of uncritically accepting TI public relations (de Andrade et al., 2017; Lucherini, 2018). The difficulty of maintaining a neutral position on such a divisive and controversial topic became apparent during our own review process, which highlighted the need to attend sufficiently to the social and public health costs of corporate political activity (Ulucanlar, Fooks, & Gilmore, 2016).

At the heart of this confusion and disruption is the product itself. Many – if not most – in public health would agree that ANDS are significantly less harmful than combustibles. This has never happened before. ANDS, which include products produced by the TI, are now being actively recommended both by UK government health services (NHS, 2018) and Europe’s biggest cancer charity (Cancer Research UK, 2017) to help people stop smoking. The TI meanwhile is enjoying a gamut of new business opportunities. It can now diversify in a way that it has been unable to do in its 150-year existence. It can plan long-term beyond the impasse of a lethal product and is even able to contemplate reintroduction to the respectable business community.

Yet, as for any corporation, the fiduciary imperative remains. The TI, whatever the improvements in its products, will always prioritise decisions on the basis of profitability and corporate growth (Branston & Sweanor, 2015). From a critical public health perspective, the concern here is that the interests of the investors will always be preferred if the ‘win-win-win’ for society, customers, and shareholders hits problems. As both independent and TI respondents noted in our study, profits are the arbiter of success, and any health or social benefits are accidental.

The independent sector is not dominated by corporations, but, nonetheless, market imperatives apply. So, while health and social benefits are, for some companies at least, core business strategies and these can drive innovation in the vaping sector, commercial sustainability also depends on financial outcomes. At the time of writing, the independent product Juul was dominating the mass-manufactured e-cigarette market, holding more than 70% of US sales, and ahead of ANDS offerings from Big Tobacco (analyst, personal communication, 27 July 2018). This, amid concerns of Juul’s youth appeal and use (Halliwell & Saker-Clark, 2018).

It remains to be seen whether this product specifically, or disruptive technology in this space, will ‘lead to more than changes in industries and consumer behaviour’ and genuinely challenge ‘existing power relationships and established wisdom’ (Stimson, Thom, & Costall, 2014, p. 654). Alternatively, perhaps, it is just a matter of time before one of the big players will acquire the ‘nascent interloper’ (Int13) as has happened in the past (Bauld et al., 2016). Tobacco companies certainly have the means to take over competitors when threatened. The Marlboro brand alone was valued at US$24.1 billion in 2017 (Forbes, 2018). However, Juul was recently valued at US$15 billion just three years after arriving on the market which suggests that it could be financially self-sufficient in relation to investment or acquisition from major tobacco players (Financial Times, 2018). It is worth noting though that the global cigarette industry was valued at more than US$683 billion in 2016 compared to the global e-cigarette market which was worth US$11.92 billion (Campaign for Tobacco Free Kids, 2017; TechNavio, 2016), although this is expected to reach US$48 billion by 2023 (PRNewswire, 2018).

Disruptive innovations are not unique to tobacco control. Equivalent technological solutions – with concomitant business opportunities − are emerging in the obesity and alcohol fields (Crino, Sacks, Vandevijvere, Swinburn, & Neal, 2015), and there are those in public health who welcome these moves (Derricott, 2015). Arguably, also, it is a predictable development in our liberalised market system, where consumption is the route to contentment, and every problem becomes a potential new product or service development opportunity.

When problems arise which cannot be solved by consumption, however, the market finds it much more difficult to respond. Climate change is the obvious and pressing example here. As climate scientist Stephen Emmott explains, ‘we need to consume less. A lot less. Less food, less energy, less stuff. Fewer cars, electric cars, cotton T-shirts, laptops, mobile phone upgrades. Far fewer’ (Emmott, 2013, p. 184). Such shrinkage is anathema to corporate capitalism (Jackson, 2009).

Thus, the problems we face are not just those of a few aberrant markets, such as tobacco, alcohol or unhealthy food, or the occasional misbehaving corporation, but of an economic system that is predicated on ever-increasing consumption and perpetual growth. This is at odds with the reality of a finite planet and suggests that, rather than just looking to the market for potential solutions, such as e-cigarettes, there is also a need to critique its failings and seek ‘coherent alternative[s]’ (Fisher, 2009).

## Note

1. During the review process for this paper, PMI announced plans to go ‘tobacco-free’ and established a US$1 billion foundation for a smoke-free world. See https://www.pmi.com/who-we-are/designing-a-smoke-free-future.

## Supplementary material

Supplemental Material - Appendices
