# Supplementary material for: Hostage to fortune: an empirical study of the tobacco industry’s business strategies since the advent of e-cigarettes
Source: Crit Public Health. 2018 Dec 18;30(3):280–93. doi: 10.1080/09581596.2018.1552778 (PMC7254526; doi:10.1080/09581596.2018.1552778)
Supplement: Supplemental Material - Appendices [file CCPH_A_1552778_SM6000.pdf]

Supplemental file to "Hostage to Fortune: An Empirical Study of the Tobacco Industry's Business Strategies Since the Advent of E-Cigarettes"

Contents

- Appendix 1: Databases, websites (subscription and open-access) and print publications searched
- Appendix 2: Scottish Government consultation responses reflecting potential strategic business opportunities
- Appendix 3: Multiple coding strategy
- Appendix 4: Codes used in the business strategies data analysis
- Appendix 5: Thematic network

Appendix 1: Databases, websites (subscription and open-access) and print publications searched

**Databases:**<br>**WARC** (World Advertising Research Center) (subscription) 20.11.15<br>**Euromonitor International's Passport** (subscription) 20.11.15 (and 4.2.16 update)<br>**Nexis** 3.2.16<br>1. Market Insight Search<br>2. Powersearch of UK 'Industries and Markets' (Market research reports and Trade press) and 'Companies and Organisations' sources<br>3. Powersearch of UK News for Industry 'Banking and Finance'<br>4. Powersearch of UK News for Industry 'Marketing and Advertising'<br>5. Powersearch of All English Language News<br>[Example search string for Nexis: ecig! OR efag! OR eshish! OR e-hookah! OR e-cig! OR e-fag! OR e-shish! OR ehookah! OR (cig! w/4 (electr! OR battery OR recharge! OR cartridge!)) OR (fag! w/4 (electr! OR battery OR recharge! OR cartridge!)) OR (shish! w/4 (electr! OR battery OR recharge! OR cartridge!)) OR (hookah! w/4 (electr! OR battery OR recharge! OR cartridge!)) OR (vap! w/s (cig! OR fag! OR tobacco! OR nicot! OR smok!))<br>**REUTERS** 3.2.16<br>**PRNewswire** 4.2.16<br>**ECigIntelligence** 5.2.16
**Websites:**<br>Electronic Cigarette Industry Trade Association<br>Irish Vape Vendors Association<br>Medicines and Healthcare products Regulatory Agency<br>**Google searches (e.g.):**<br>e-cigarettes business strategy<br>e-cigarettes forecast business
**Hand-search publications:** Apr '15-Mar '16<br>*Tobacco Journal International*<br>*Tobacco Reporter*<br>*Vapor Voice*

Appendix 2: Scottish Government consultation responses reflecting potential strategic business opportunities

Consultation response from:	Responses reflecting potential strategic business opportunities:<br>1. Diversification / long-term strategic opportunities<br>2. Profitability/lack of taxation<br>3. Lead safety/science<br>4. Route into regulation/work with regulators<br>5. Rebuild corporate reputations/guard against litigation
**Fontem Ventures**<br><br>**Wholly-owned by Imperial Tobacco Group**	**2. Profitability:**<br>Advocate a flat-rate tax for e-cigs and those without nicotine should not be taxed.<br>**4. Regulation:**<br>**Domestic advertising and promotion protection** Oppose a blanket ban to allow awareness-raising, communication of factual info and compete as an alternative to tobacco products. However, should not target under-age or non-smokers. CAP rules provide an appropriate model for regulation.<br>**Additional regulations** Regulation should stipulate anti brand-stretching and youth protection measures for all advertising formats. E-cigs which are licensed as medicinal devices should not be subject to specific regulation but comply with regulation for medical devices. However, "heat-not-burn" products should fall within current scope of legislation as they contain tobacco.<br>**5. Reputation:**<br>Contribution to public health is a key strategy.
**Japan Tobacco International (JTI)**<br><br>**JTI, a leading tobacco manufacturer, acquired Zandera Ltd in 2014, one of the UK's largest e-cig companies. E-lites is the main brand and was seen as a logical extension to the JTI portfolio.**	**4. Regulation:**<br>**Domestic advertising and promotion protection** Regulators should avoid excessive regulation that prevents adult consumers from choosing these products. JTI does not market e-cigs or other nicotine-containing products to minors or non-smokers.<br>**Additional regulations** Support reasonable and proportionate regulation − no additional steps required.<br>**Smokefree NHS grounds** Adults should be able to make their own informed choices. Not legitimate to treat the use of tobacco as abnormal, unacceptable or tainted. There is a high awareness of the danger of smoking, particularly within the health profession.<br>**Smokefree tobacco family areas** Would support info campaigns rather than bans, which engage smokers rather than stigmatise.<br>**5. Reputation:**<br>Proud of training schemes for retailers on age-restricted products − should apply the Challenge 25 rule voluntarily. Scottish Government should conduct an Impact Assessment to quantify dampening effect of restrictions on retailers, distributors and manufacturers of e-cigs. Contribution to public health is a key strategy.
**Blu e-cigs (UK) Ltd**<br><br>**Lorillard Inc. parent company**	**1. Diversification/Long term:**<br>Not marketing as reduced harm or reduced risk products, but as an alternative to tobacco products.<br>**3. Safety/science:**<br>Key strategy is advancing scientific research into safety and efficacy of e-cigs to compete with NRT.

	Refers to studies carried out by Lorillard Inc. with regards to e-cigs in public places.<br>**4. Regulation:**<br>**E-cigs in public places** Employers and organisations should be able to adopt their own policies. Refers to studies carried out by Lorillard Inc.<br>**Additional regulations** No, not in addition to TPD. Disproportionate restrictions on marketing would create hurdles to market entry; restrict consumer choice; increase in illicit trade and unsafe products; restrict product innovation; and lead to Scotland being out of sync with rest of UK.<br>**5. Reputation:**<br>Contribution to public health is a key strategy.<br>Welcomes Scottish Government's willingness to engage with business.
**Nicocigs**<br><br>**Nicocigs was acquired by Phillip Morris Intl in June 2014**	**1. Diversification/Long term:**<br>Excessive regulation will restrict our commercial freedom to communicate with adult smokers. This will reduce the appeal of e-cigs and thus reduce number of smokers switching from combustibles.<br>**3. Safety/Science:**<br>E-cigs less hazardous for by-standers. Restrictions in public places should be considered in light of scientific evidence; the need to encourage switching; desire not to expose minors; and where e-cigs could present a hazard.<br>**4. Regulation:**<br>**Domestic advertising and promotion protection** Young people should be protected, but not any further than the TPD regulations. Adult smokers should receive info on e-cigs and e-cig businesses have commercial freedom to compete. E-cigs should continue to be advertised and promoted at POS, and places that do not have any particular appeal to minors.<br>**Additional regulations** Products should carry a health warning that they contain nicotine, a highly addictive substance. Need to strike a balance between protecting minors and non-smokers from exposure to advertising and the need to ensure adult smokers have awareness of the potential benefits of these products.<br>**E-cigs in public places** Organisations should be free to impose their own policy.<br>**5. Reputation:**<br>Public health is a key strategy.
**Imperial Tobacco Ltd**	**1. Diversification/Long term:**<br>Already extensive tobacco control measures, however, and should be an evaluation of their effectiveness before further restrictions imposed. E-cigs fundamentally different.<br>**4. Regulation:**<br>**Additional regulations** Brand-stretching and events sponsorship with a domestic setting should be regulated. Exemptions should apply to flavours as a key component of e-cigs, but should not be particularly appealing to young people. Advertising of e-cigs which are licensed as medicinal devices should not be subject to e-cig specific regulation.
**Nicoventures**<br><br>**Nicoventures is a division of BAT, focusing on development and**	**1. Diversification/Long term:**<br>Bringing e-cigs under remit of smoke-free legislation would inhibit switching.<br>**2. Profitability:**<br>Whilst regulation should be introduced to ensure product quality and safety, they should not stifle innovation and investment. A need for a retailer register and further restrictions on advertising would have financial implications on the industry. Would result in reduced investment. Do not introduce additional obstacles for a category which has potential public health benefits.

**production of nicotine products.**	**3. Safety/Science:**<br>Product quality and safety also a key strategy.<br>**4. Regulation:**<br>**Domestic advertising and promotion protection** Yes. However, young people will be exposed to some e-cig advertising. Welcome BCAP and CAP measures to protect young people and non-smokers.<br>**Additional regulations** Adults have a right to be informed about alternatives to smoking. No evidence they are a gateway to smoking.<br>**E-cigs in public places** Bringing e-cigs under remit of smoke-free legislation would be damaging to public health, as it would inhibit switching. However, it should be prerogative of business owner as to whether allowed or not.<br>**5. Reputation:**<br>Public health is a key strategy.
**FOREST**<br><br>**Lobbying body for right to enjoy smoking. Supported by BAT, Imperial Tobacco Ltd and Gallaher Ltd but views expressed are those of FOREST alone.**	**4. Regulation:**<br>**Additional regulations** Excessive regulation would compromise ability of businesses to market a product that could have a significant impact on public health, as it would reduce rate of switch. E-cigs satisfy a desire for nicotine which is no more harmful than caffeine.<br>**E-cigs in public places** Yes, no evidence of any risk from passive smoking from e-cigs. Counter-productive to the aims of tobacco control. We support consumer choice and evidence-based policy. The threat of a comprehensive ban on the use of e-cigs in enclosed public places is worse than the smoking ban for smokers who have taken the decision to cut down or quit smoking with the aid of e-cigs.<br>**Smokefree NHS grounds** comprehensive ban is disproportionate to the problem.<br>**Smokefree tobacco family areas** Whilst we do not condone smoking in children's outdoor areas, we believe a national ban is heavy-handed. In general, adults know how to behave when smoking around children.

Notes: *These are selected abbreviated or paraphrased texts from the organisations' submissions to the Scottish Government's 2014-15 consultation on electronic cigarettes and tobacco control. For the entire submissions, please see: Scottish Government (2015, May 6).* Reponses to the Scottish Government's consultation on electronic cigarettes and tobacco control which ran from 10 October 2014 – 2 January 2015. *Retrieved from http://www.gov.scot/Publications/2015/05/5127/0.*
Abbreviations: *BAT British American Tobacco, BCAP Broadcast Committee of Advertising Practice, CAP Committee of Advertising Practice, POS point-of-sale, TPD Tobacco Products Directive.*

Appendix 3: Multiple coding strategy (R=Researcher)

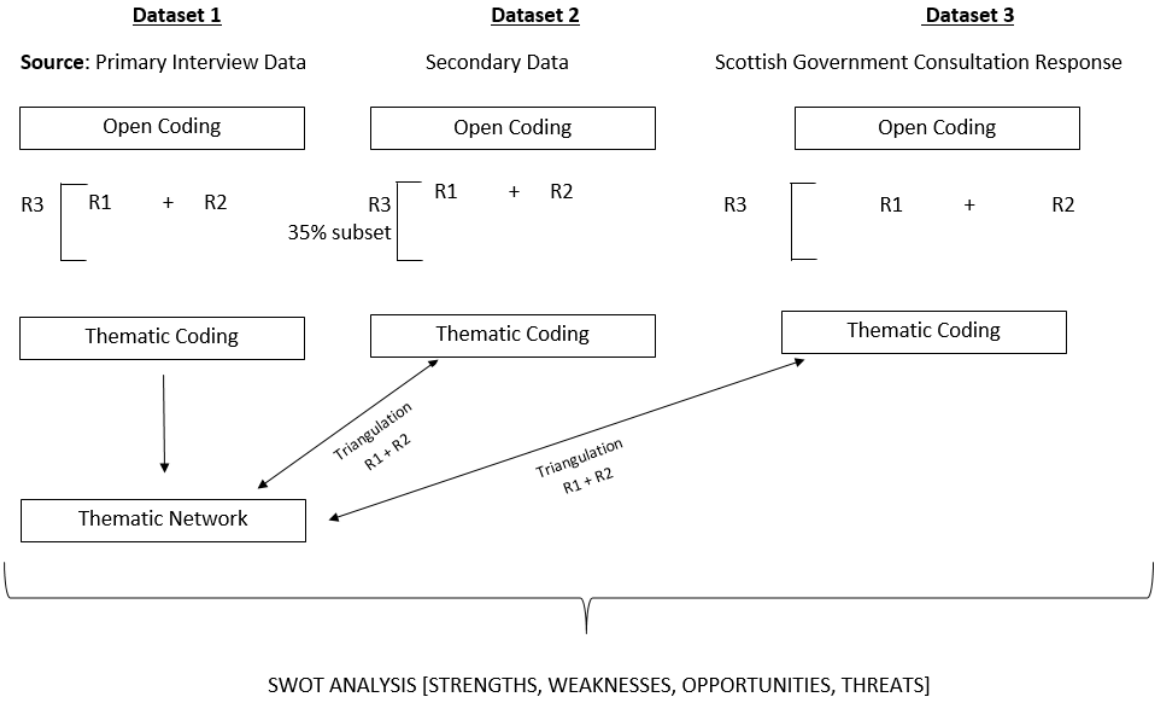

Appendix 4: Codes used in the business strategies data analysis

Dataset 1: interview transcripts (primary data):

Open coding

Tobacco Industry

1. Threats to opportunities
2. Sustainability
3. Long-term use
4. TI is not a homogenous entity
5. Different strategies and goals, motivations
6. React and adapt
7. Emerging vs 'dying' markets
8. Transparency
9. Shareholder value
10. New tobacco execs, new category, new approach
11. Market share
12. Full portfolio
13. Safety, high quality standards
14. Clear communication of risk
15. Regulation [to protect the companies vs innovation]
16. Science
17. Protect the cigarette
18. Complete transformation
19. Competitive advantage
20. Scale and distribution
21. Keep the consumer, meet the consumer's needs
22. Legal
23. Consolidation [vs innovation]
24. The Apple/iPhone model: closed vs open systems – brand creation [linked to heat not burn]
25. Grow and compete / expand the market

Independent

1. End of combustibles
2. Opportunity for profit
3. Open system / individualised product / understanding the consumer
4. Survival
5. Retail model
6. Innovation
7. Lobbying
8. Build a brand/ credibility

Other

1. Battlefield OR Battles and discomfort [and confusion] [and ideology] – between PH groups, between TI companies, within TI companies / industry is not an homogenous mass
2. Confusion [PH]
3. Terminology
4. Ideology
5. Vested interests

Thematic coding

Tobacco Industry Business Strategies

Shareholder value and maximising profits

1. Protecting the cigarette market to grow the ANDs market
2. Threat into opportunity: react and adapt

3. Keep the consumer
4. Diversification: a full portfolio
5. Sustainability
6. Scale and distribution
7. The “Apple model” (closed systems) versus open systems
8. Consolidation or Research & Development (R&D)
9. Science
10. Transparency and a clear communication of risk
11. Safety, quality and high product standards
12. Regulatory pathways
13. Litigation
14. Competitive advantage
15. Emerging vs ‘dying’ markets

<u>Independent Business Strategies</u>

1. End of combustibles
2. Individualised products
3. Retail business model
4. Organic business model / shared engagement
5. Opportunity for profit / survival
6. Innovation versus regulation

Abbreviations: TI tobacco industry, PH public health

Dataset two: text documents (secondary data)

Open coding:

<u>Company</u>

independent, tobacco industry, unclear or don’t know, unspecified ‘e-cigarette’ industry

<u>Names</u>

named brands, companies and trade organisations

<u>Product categories</u>

cig-a-likes, e-liquids, tank system devices, heat-not-burn items

<u>Statistics</u>

market data or statistics

<u>NRT</u>

negative impact on traditional nicotine replacement therapies (NRT)

Thematic coding:

<u>Business strategies</u>

1. Business approach
2. Health claim
3. Position on harm reduction
4. Targeting strategies
5. Marketing plan (including distribution, packaging, pricing, product development, promotion, other)

<u>Other business strategies</u>

product labelling, advertorials

Dataset three: Scottish Government consultation documents (secondary data)

Open coding:

6. Age restrictions on e-cigarettes
7. Domestic advertising and promotion protection
8. Additional regulations
9. Inclusion of e-cigarettes on Tobacco Register?
10. Permit e-cigarettes in public places?

11. Smoke-free NHS grounds?
12. Smoke-free tobacco family area?
13. ‘Challenge 25’ for e-cigarettes and tobacco products?
14. Business and regulatory impacts considerations
15. Business strategies

Thematic coding:

Business strategies

1. Scale and distribution
2. Consolidation or R&D
3. Science
4. Transparency and a clear communication of risk
5. Safety, quality, high product standards
6. Regulatory pathways

Appendix 5: Thematic network

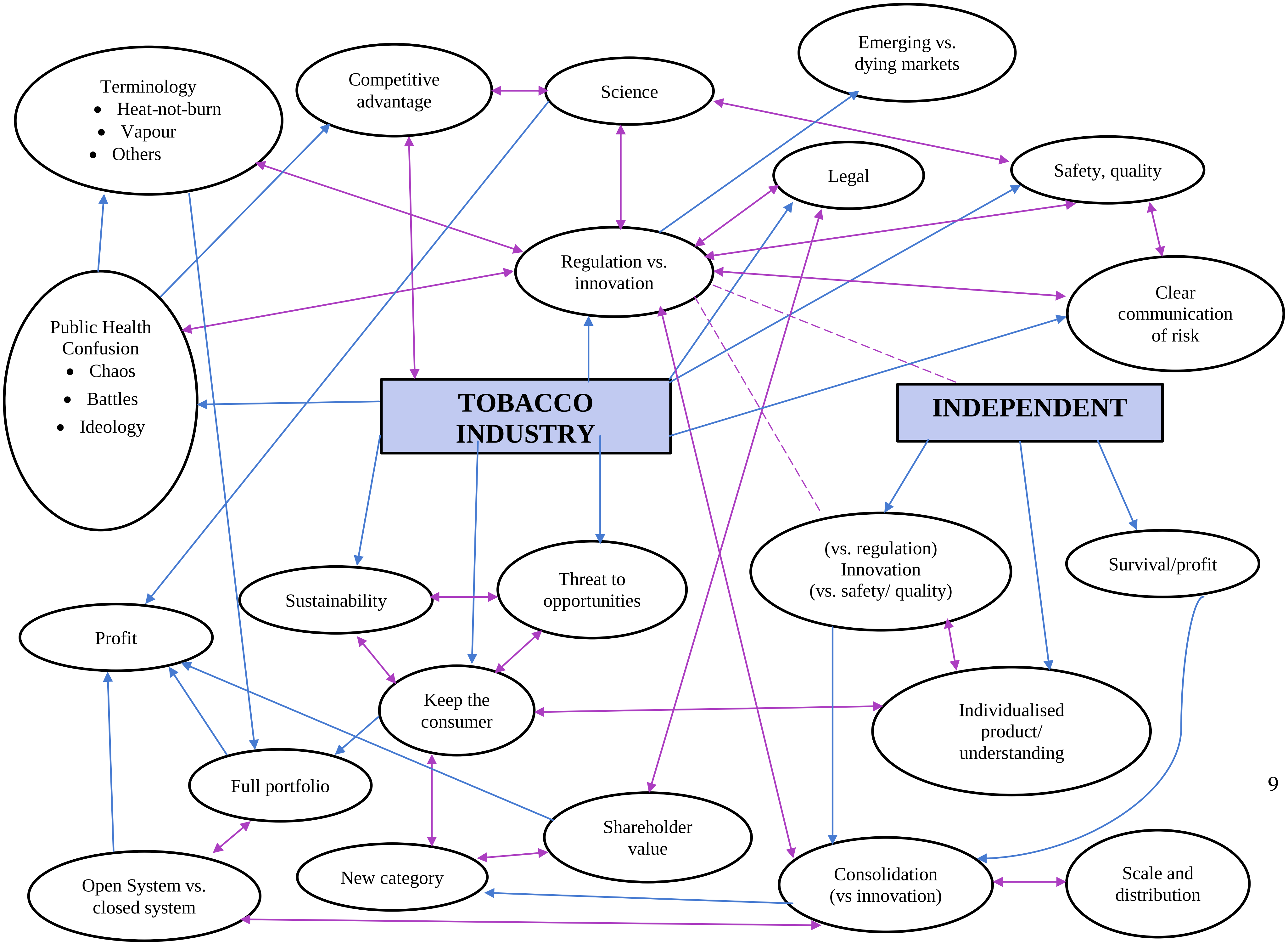

Terminology
Heat-not-burn
Vapour
Others
Competitive advantage
Science
Emerging vs. dying markets
Legal
Safety, quality
Regulation vs. innovation
Clear communication of risk
Public Health Confusion
Chaos
Battles
Ideology
TOBACCO INDUSTRY
INDEPENDENT
(vs. regulation) Innovation (vs. safety/ quality)
Survival/profit
Sustainability
Threat to opportunities
Profit
Keep the consumer
Individualised product/ understanding
Full portfolio
Shareholder value
Open System vs. closed system
New category
Consolidation (vs innovation)
Scale and distribution
